# The Untapped Genetic Reservoir: The Past, Current, and Future Applications of the Wild Soybean (*Glycine soja*)

**DOI:** 10.3389/fpls.2018.00949

**Published:** 2018-07-09

**Authors:** Janice Kofsky, Hengyou Zhang, Bao-Hua Song

**Affiliations:** Department of Biological Sciences, University of North Carolina at Charlotte, Charlotte, NC, United States

**Keywords:** wild soybean, *Glycine soja*, crop wild relative, genomics and genetics, biotechnology, crop improvement, stress response and tolerance

## Abstract

There is a considerable demand for crop improvement, especially considering the increasing growth of world population, continuing climatic fluctuations, and rapidly evolving plant pests and pathogens. Crop wild relatives hold great potential in providing beneficial alleles for crop improvement. Wild soybean, *Glycine soja* (Siebold & Zucc.), the wild ancestor to the domesticated soybean (*Glycine max* (L.) Merr.), harbors a high level of genetic variation. Research on *G. soja* has been largely devoted to understanding the domestication history of the soybean, while little effort has been made to explore its genetic diversity for crop improvement. High genomic diversity and expanded traits make *G. soja* populations an excellent source for soybean improvement. This review summarizes recent successful research examples of applying wild soybeans in dissecting the genetic basis of various traits, with a focus on abiotic/biotic stress tolerance and resistance. We also discuss the limitations of using *G. soja*. Perspective future research is proposed, including the application of advanced biotechnology and emerging genomic data to further utilize the wild soybean to counterbalance the rising demand for superior crops. We proposed there is an urgent need for international collaboration on germplasm collection, resource sharing, and conservation. We hope to use the wild soybean as an example to promote the exploration and use of wild resources for crop improvement in order to meet future food requirements.

## Soybean and wild soybean

Cultivated soybean (*Glycine max* (L.) Merr.) is an economically important crop grown world-wide with diverse uses in oil and protein consumption for human and livestock, as well as feedstock for biofuel production. Cultivated soybean produces two-thirds of the world's protein meal, and is the leading producer of oilseed (Oecd Food and Nations AOOTU, 2016). With the growing world population, the yield for soybean is at a current deficit of 1.2% annual production per year (Ray et al., 2013). Although soybean yield per acre has increased by approximately 40% in the last quarter century and production has more than doubled (1990–2015) (USDA, 2016), current soybean yield potential is restricted by the narrow genetic variation in *G. max*, hindering the potential for breeding soybean varieties with high environmental stress tolerance and resistance traits. These concerns regarding the current cultivars are increasing because of the ecological changes caused by climate fluctuations and other factors such as expanded drought or saline environments. Meanwhile, the rapid evolution of pests and pathogen is posing difficult challenges to soybean production. Because of the aforementioned factors, there is an urgent need to use the untapped genetic resources from the wild relatives of cultivated soybean to improve soybean production.

**Figure 1:**
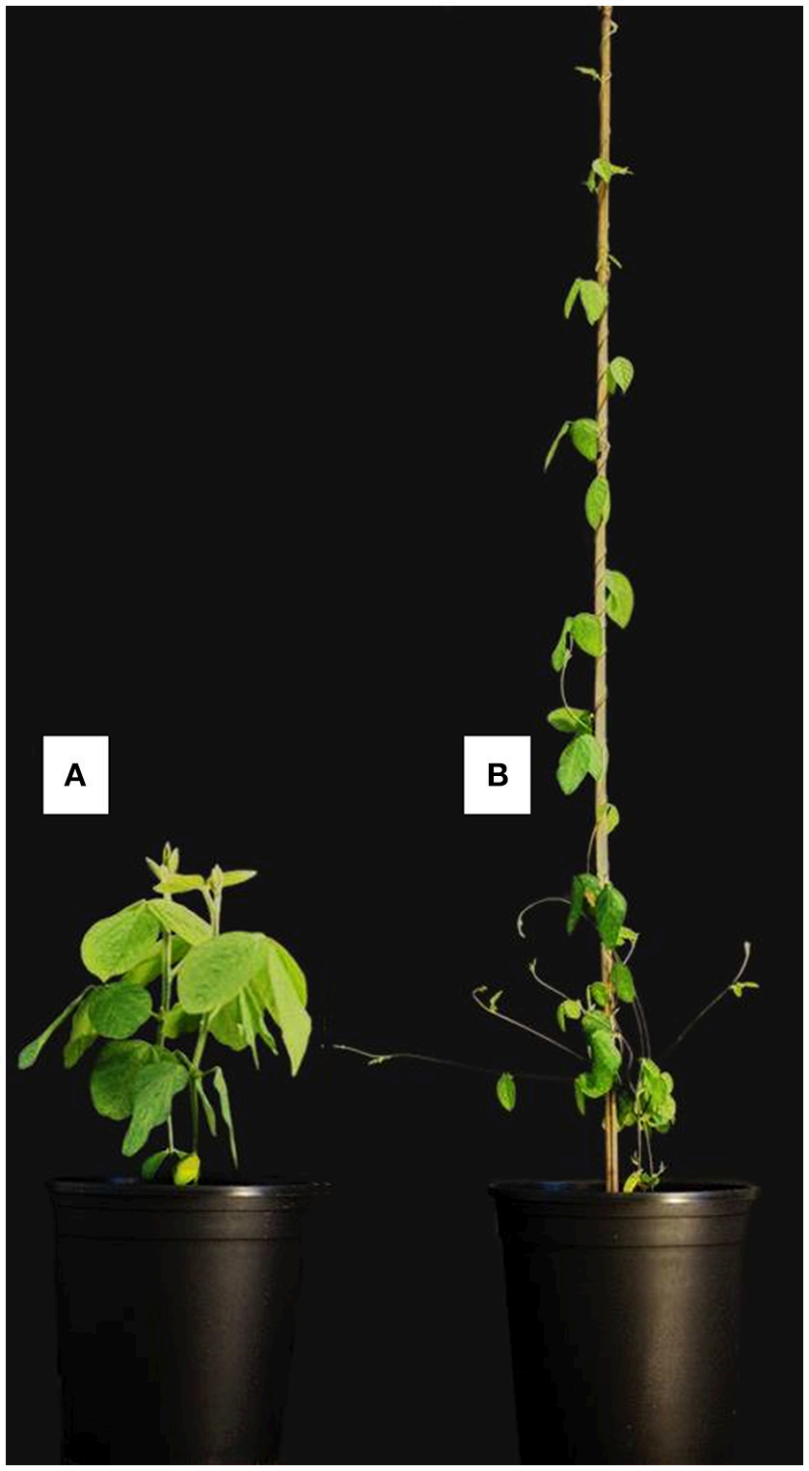
Illustration of phenotypic comparison between *G. max* **(A)** and *G. soja* **(B)** (Photo taken by J. Kofsky).

Wild soybean, *Glycine soja* (Siebold & Zucc.), is the wild ancestor of the domesticated soybean, *G. max*. *Glycine soja* is native to East Asia with a broad geographic range, from East Russia to South China, and the species can grow in diverse habitats. Though *G. soja* and *G. max* are different in many phenotypic characteristics (Figure 1), they have the same number of chromosomes (2n = 40), exhibit normal meiotic chromosome pairing, and are cross-compatible (Carter et al., 2004). Also, *G. soja* was found to harbor a high level of genetic diversity, which might have been lost in *G. max* during their domestication and improvement (Hyten et al., 2006). Considering the higher level of genetic diversity retained in *G. soja*, as well as its adaptations to harsh environments, *G. soja* holds great potential to improve its agriculturally important domesticated relative, beyond what is currently known (Stupar, 2010; Qiu et al., 2013; Qi et al., 2014; Zhang et al., 2017d). Research on *G. soja* has been largely devoted to understanding the domestication history of the soybean, with comparatively little effort made to use it as a genetic reservoir for soybean improvement (Kim et al., 2011; Qi et al., 2014; Zhang et al., 2016). In this review, we will discuss genomic diversity and the current research in *G. soja*, and we present a potential step forward in our application of *G. soja* to improve *G. max*.

## Domestication history

Domestication of *G. soja* is reported to have occurred around 6,000-9,000 years ago in regions along the Yellow River or Huang-Huai Valley in Central China (Carter et al., 2004; Li et al., 2010; Han et al., 2016), resulting in landraces of *G. max*, and with further selection the modern (elite) cultivated material. However, the history of *G. soja* in relation to *G. max* is far more complex, with varying contradictory hypotheses. It is likely that the domestication process happened over a long period of time, allowing for frequent introgressions between the wild and cultivated populations during this time (Wang and Li, 2013; Qiu et al., 2014). Although an opposing study of candidate domestication regions in Korean wild soybeans suggests a single selective sweep, detecting no evidence for multiple domestication events in East Asia (Chung et al., 2014). A summation of the contradictory hypotheses of soybean origin and domestication was recently presented in a review of the domestication history (Sedivy et al., 2017), in which three overarching hypotheses were provided: the single origin hypothesis, the multiple origin hypothesis, and the complex hypothesis. The single origin hypothesis states that *G. max* was diverged from *G. soja* from a single domestication event in Central China, no earlier than 9,000 years ago, supported by the observation that all the selected domesticated soybeans were clustered together by analyzing whole-genome SNPs of 302 wild, landrace and cultivated soybeans (Zhou et al., 2015b). The multiple origin hypothesis states that *G. max* was domesticated from *G. soja* during multiple events between 5,000 and 9,000 years ago. The complex hypothesis incorporates the results from two recent studies (Kim et al., 2010; Li et al., 2014), with a *G. soja*/*G. max* complex first diverging before multiple domestication events. The estimated age of the *G. soja*/*G. max* complex is 0.27 million years ago (MYA) (Kim et al., 2010) by whole genome comparison of one wild soybean ecotype to one soybean cultivar, or 0.8 MYA (Li et al., 2014) by pan-genome comparison of 7 wild soybean ecotypes. In this last hypothesis, the domestication would have stemmed from an already diverged *G. soja*/*G. max* complex (Sedivy et al., 2017). Individuals from either *soja* or *max* subpopulation were closely clustered based on their geographic origins (Zhang et al., 2015, 2016). A possible explanation could be that the early-domesticated *G. soja* or *G. soja/G. max* complex spread from China to Korean and Japan, and subsequently underwent varying degrees of domestication to meet local needs. Nevertheless, it is widely accepted that *G. max* was created from *G. soja or G. soja*/*G. max* complex through a long, slow, and complex domestication process by countless independent efforts (Sedivy et al., 2017).

## Advantages of using wild soybean

*G. soja* gene pool is indisputably more diverse than *G. max* due to artificial selection during domestication and continued loss due to modern breeding practices. A comparison of 102 gene sequences from 26 *G. soja*, 52 landraces, 17 North American ancestors, and 25 elite cultivar isolates suggested that the most significant loss in genetic diversity occurred during the domestication bottleneck, and a secondary loss of diversity during modern breeding. The domestication bottleneck has resulted in an 81% loss of rare alleles, 60% gene allele frequency change, and almost halving the nucleotide diversity (π) from *G. soja* (π = 2.47 × 10^−3^) to landrace (π = 1.47 × 10^−3^). After domestication, intense selection toward the elite cultivars (π = 1.17 x 10^−3^) resulted in an additional loss of 23% nucleotide diversity (π), and a 21% loss of rare alleles (Hyten et al., 2006). These artificial selection processes resulted in morphological differences of many agriculturally-important traits between *G. max* and *G. soja*, such as pod shattering resistance (Dong et al., 2014), determinate growth habit (Tian et al., 2010), and seed-related traits (Zhou et al., 2015a). Directional selection during modern soybean breeding practice also reduced the genetic diversity surrounding the regions conferring these agriculturally important traits, known as selective sweep. A recent genome-wide sequencing analysis showed that half of the resistance-related genes/loci in *G. soja* were not found in landraces or the domesticated soybean (Zhou et al., 2015b). Thus, modern breeding practices have further shrunk the gene pool by selecting from a small fraction of landraces to produce elite cultivars (Song et al., 2015). These observations suggest that the morphological characters of the elite cultivars are genetically controlled by a combined effect of these genomic regions that were selectively swept or lost during domestication and artificial selection. It is possible that reduced genetic diversity in elite cultivars can be significantly increased by introgression of the favorable variation in *G. soja* in modern breeding program, while additional efforts are needed to balance the selection of agriculturally and adaptively important traits.

**Figure 2:**
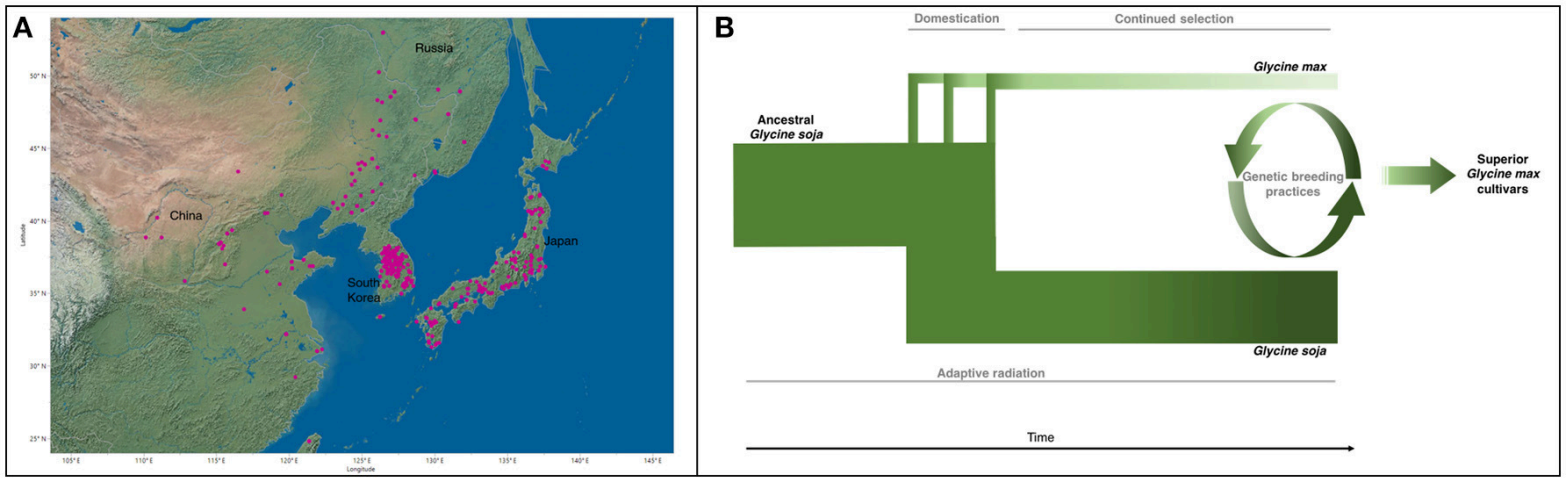
**(A)** Geographic distribution of all *G. soja* ecotypes with identified locations (Geographic location data were retrieved from URL: https://training.ars-grin.gov/gringlobal/search.aspx). **(B)** *G. max* diverged from ancestral *G. soja* as a result of multiple domestication events. *G. max* then underwent continued artificial selection for traits of agronomic importance, further reducing the genetic diversity found in *G. max*. During this time, *G. soja* continued to adapt to its various environments, maintaining and potentially increasing genetic variability. Genetic breeding practices incorporate select components from the *G. soja* gene pool to improve modern cultivars, developing superior *G. max* cultivars. Width and color represent genetic diversity (Sedivy et al., 2017).

Natural populations of *G. soja* were strongly influenced by climatic fluctuations. The Quaternary glaciation around 2 MYA resulted in the shrinking or distinction of many plant populations (Qiu et al., 2011). Bottlenecks of most natural populations resulted in loss of genetic diversity within populations and differentiation among populations by genetic drift and environmental selection (Guo et al., 2012; Li et al., 2014; Leamy et al., 2016). It is likely that *G. soja* populations then expanded rapidly through Asia (Figure 2A) after glaciation due to its high adaptability (Sakai et al., 2003; Wang et al., 2016). This evolutionary history accounts for the wide range of genetic and phenotypic diversity found among *G. soja* populations. The population size of *G. soja* is expanding, while *G. max* has been relatively constant (Wang et al., 2015), with correlated disparity in genetic diversity seen in Figure 2B. Adaptations to local environments has enabled *G. soja* to develop sophisticated mechanisms to tolerate many biotic and abiotic stressors. Many of the resistance-causal genes have been found to exist in manners of copy number variations (CNVs), such as *rhg1* conferring soybean cyst nematode (SCN) resistance (Cook et al., 2012). Environmental/niche isolation played a stronger role than isolation by geographic distance in the genetic differentiation of *G. soja*, suggesting the presence of many environmentally tailored adaptations in natural populations (He et al., 2016; Leamy et al., 2016). Lee et al. (2015) found that the origin of the tandem duplication of the 31.2-kb segment at the *rhg1* locus (Cook et al., 2012), a major QTL conferring SCN resistance, occurred prior to the divergence of *G. max* and *G. soja* or the formation of the ancestor of *G. max*/*G. soja* complex, implying that the copy number variation in *rhg1* evolved from a *G. soja* population in East Asia, where the SCN populations most likely originated (Tylka and Marett, 2014).

**Figure 3:**
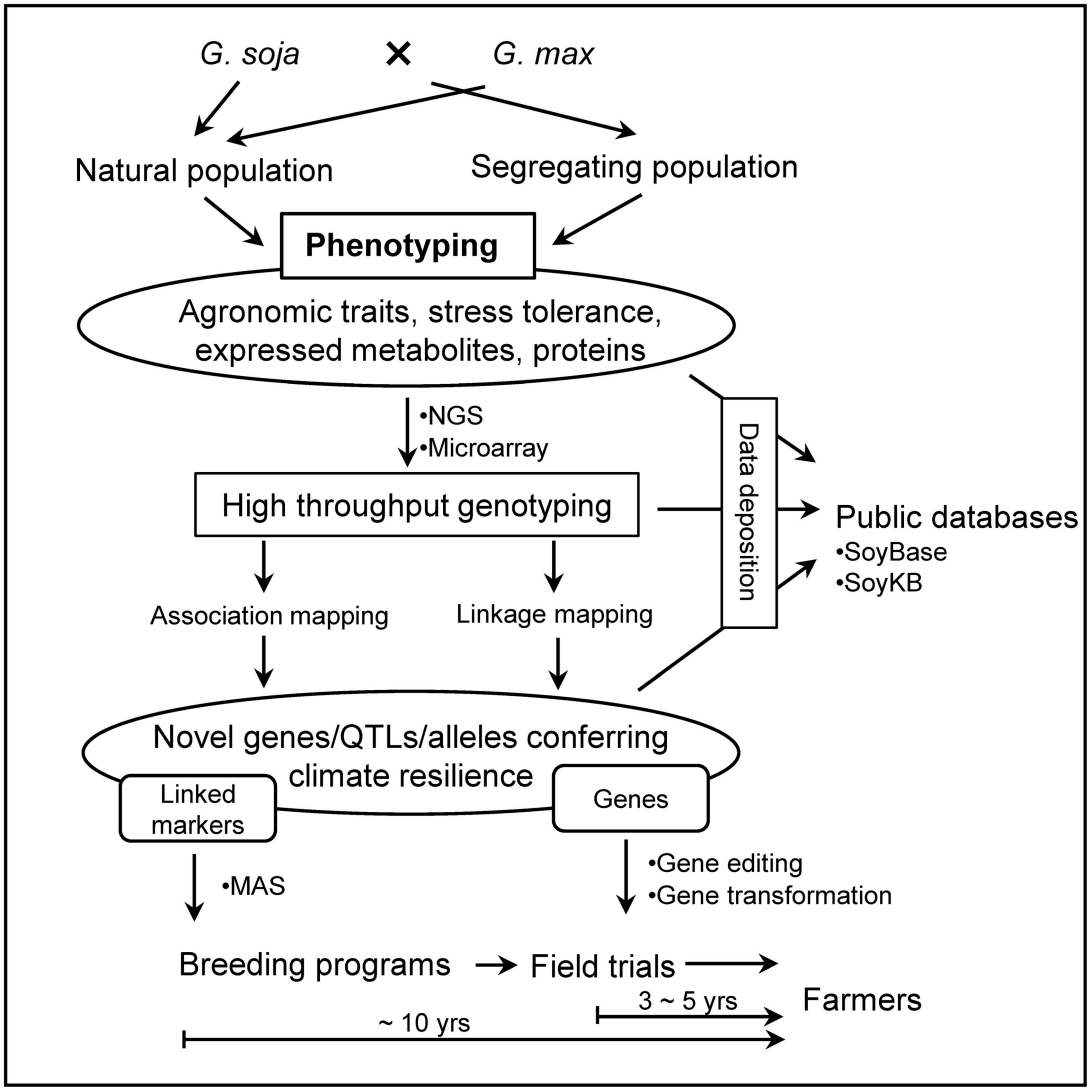
Research pipeline of crop improvement of *G. max* using *G. soja* (Fernie and Schauer, 2009).

The concept of revitalizing the cultivated gene pool with a wild progenitor species has been well applied in many crops. Plant domestication limits the range of obtainable phenotypes by artificial selection, often removing unforeseen traits of interest down the line. For example, during the 1970s, commercial corn crops (*Zea mays* L.) were devastated by blight affecting as much as 50% of the yield in the United States, until blight-resistant alleles from the wild relative (Mexican maize, *Tripascum dactyloides* L.) were introduced into the domesticated population (Maxted and Kell, 2009). Examples of the use of crop wild relatives to improve abiotic stress tolerance, biotic stress resistance, and agronomic traits of cultivated crops has been seen in major crops: rice, barley, wheat, tomato, potato, and peanut (Zhang et al., 2017c). The development of superior soybean cultivars by incorporating genes/alleles from *G. soja* (Figures 2B, 3) is a promising and environmentally friendly solution for soybean improvement, moderating or negating the need for pesticides and fertilizers.

## Genomic resources and discoveries from *G. SOJA*

In 2010, a *G. soja* ecotype (IT182932) and 17 additional ecotypes were resequenced and assembled using *G. max* as a reference, serving as the first reported whole-genome sequences of *G. soja* (Kim et al., 2010; Lam et al., 2010). Structural characteristics of the genome were reported 2 years later, creating a physical map, useful for investigating genome architectural differences between *G. max* and *G. soja* (Ha et al., 2012). The development of the SoySNP50K iSelect Illumina BeadChip and the release of up to 50,000 genome-wide single nucleotide polymorphism (SNPs) in 2013 further promoted the studies and applications of wild soybean (Song et al., 2013). Information regarding soybean research, including all SNPs for over 20,000 of USDA soybean accessions, is publically available at the SoyBase database (https://soybase.org/), serving as an excellent resource for various studies, such as genomic diversity and gene discovery in wild soybeans.

Research on *G. soja* thus far has mostly focused on the understanding of domestication and evolutionary history of *G. soja* transition into cultivated crop. Whereas, few studies have made full use of this wild genetic reservoir. Although the utilization of the genetic diversity contained in *G. soja* is lacking, the potential of *G. soja* in soybean improvement has been gradually recognized. Table 1 summarizes the recent research and findings using *G. soja* toward soybean improvement. We discuss some representative examples in the following paragraphs and provide an in-depth consideration of these findings. *G. soja* has also been used in many studies as a negative control to identify genes for traits present in *G. max*, for example in the study of seed weight or protein content (Maughan et al., 1996; Zhou et al., 2015b). In these cases, *G. soja* gene pool that was not used as a direct source of resistance, tolerance, or improvement is not presented here as such.

**Table 1:** Findings from *G. soja* including biotic stress resistance, abiotic stress tolerance, nutrition, and yield related traits.

	**Trait**	**Gene/locus found**	**Method**	**Significance**	**Source(s)**
Biotic stress resistance	Soybean cyst nematode (*H. glycines*) resistance	Marker A245_1 (chr. 18) and Satt598 (chr. 15)	QTL mapping	QTLs associated with race 3 resistance, confirmed in a backcross population	Wang et al., 2001
		QTLs *cqSCN-006* and *cqSCN-007*. Candidate gene *Glyma.15g191200* in *cqSCN-006*	QTL mapping and positional cloning	Increased resistance when in combination with *G. max* resistance alleles (*rhg1, Rhg4, rhg1-b*). *Glyma.15g191200* encodes SNAP proteins, similar to *Rhg1* function.	Kim et al., 2011; Kim and Diers, 2013; Yu and Diers, 2017
		*MAPK* (*Glyma.18g106800*) and *CDPK* (*Glyma.18g064100*)	GWAS	New candidate genes associated with *H. glycines* type 2.5.7 resistance	Zhang et al., 2016
		*RLKs, CaMs*, JA/SA signaling genes *MAPKs, WRKYs*-	Transcriptomics	Comprehensive regulatory network conferring HG type 2.5.7 resistance in *G. soja*	Zhang and Song, 2017; Zhang et al., 2017d
	Foxglove aphid (*Aulacorthum solani*) resistance	*Raso2*	QTL mapping	Antixenosis and antibiosis resistance to foxglove aphid	Lee et al., 2015
	Aphid (*Aphis glycines*) resistance	*Rag3c* and *Rag6*	QTL mapping	Genes confer antibiosis resistance to aphids	Zhang et al., 2017f
Abiotic stress tolerance	Salt tolerance	*Ncl2*	QTL mapping	First report of salt tolerance gene in soybean separate from the S-100 derived *G. max* salt tolerance allele	Lee et al., 2009
		*GmCHX1*	Whole genome sequencing and QTL mapping	Salt tolerance via ion transporters role to maintain homeostasis during salt stress	Qi et al., 2014
		*GmTIP2;1*	Genome wide identification of aquaporins and expression analysis	Overexpression of candidate gene increases salt stress response, likely also associated with drought response	Zhang et al., 2017a
	Root architecture	*Glyma15g42220, Glyma06g46210, Glyma06g45910, Glyma06g45920*, and *Glyma07g32480* (epistatic only)	QTL mapping	SNPs in discovered genes are associated with shorter root or taproot in wild soybeans which is related to drought adaptations	Prince et al., 2015
	Alkalinity tolerance	*ALMT, LEA*, ABC transporter, *GLR, NRT*/*POT* and *SLAH* gene(s)	Transcriptomics	Genes upregulated during NaHCO_3_ stress	Zhang et al., 2016
	Drought tolerance	*GsWRKY20*	Transcriptomics and transgenic overexpression	Overexpression of *GSWRKY20* enhances tolerance to drought stress	Ning et al., 2017
Nutrition	Seed protein content	Marker pA-245 (LG C)	QTL mapping	This *G. soja* allele is associated with increased seed protein content and dominant over the *G. max* allele	Diers et al., 1992
	Seed saturated fatty acid content	SNPs ss71559532, ss715597684, ss715617910	GWAS	QTLs associated with lower palmitic acid levels	Leamy et al., 2017
		*Glyma.14G121400, Glyma.16G068500*	GWAS	Candidate genes associated with steric acid production	Leamy et al., 2017
	Seed unsaturated fatty acid content	*Glyma.16G014000, Glyma.07G112100*	GWAS	Candidate gene associated with oleic acid levels and linoleic acid levels respectively	Leamy et al., 2017
Yield	Yield	QTL on chromosome 14, Satt168 the most significant marker	QTL mapping	9% yield advantage in *G. max* individuals carrying *G. soja* QTL	Concibido et al., 2003

*LG represents linkage group, GWAS represents Genome-wide association study, chr. represents chromosome*.

### Biotic stress resistance

The soybean aphid (*Aphis glycines*) is native to Asia and was introduced to the United States in 2000. Infestations by *A. glycines* can directly affect soybean biomass and yield, and indirectly affect yield with the transmission of the soybean mosaic virus (SMV). The screening of *G. soja* for soybean aphid resistance has revealed that three *G. soja* genotypes showed resistance to the soybean aphid (Hesler, 2013). The follow-up studies identified two new QTLs, *Rag3c* and *Rag6*, related to aphid resistance using linkage mapping (Zhang et al., 2017e,f). *Rag3c* explains 12.5–22.9%, while *Rag6* explains 19.5–46.4%, of the phenotypic variance in different trials. These novel aphid-resistance gene(s) derived from *G. soja* are proven valuable in the development of aphid-resistant soybean cultivars (Zhang et al., 2017e). Map-based cloning of the two QTLs and the functional verification of candidate genes are needed to uncover the underlying mechanism. It is likely that the candidate genes within the QTL regions encode canonical nucleotide-binding site leucine-rich repeat (NBS-LRR)-containing proteins, which belong to a resistance (R) protein that has been found to be resistant to potato aphid (Rossi et al., 1998).

The first report of the genetic identification of foxglove aphid (*Aulacorthum solani*) resistance gene, *Raso2*, in *G. soja* was in 2015 (Lee et al., 2015). Up to this point, five other aphid resistance genes had been identified, with all being mapped from *G. max* cultivars. *Raso2* differs from those isolated from *G. max*, with strong antixenosis and antibiosis responses to the foxglove aphid (Lee et al., 2015), which suggests a novel mechanism of *A. solani* resistance exists in *G. soja* and merits further functional dissection.

Soybean cyst nematode (SCN) is the most devastating pest in cultivated soybeans. Breeding SCN-resistant soybean cultivars in the United States are dependent on very limited genetic sources. In 2005, 94% of the SCN-resistant cultivars grown in Illinois were sourced from a *G. max* accession (PI88788) that has shown reduced resistance to the majority of SCN populations found in the soil (Kim et al., 2011). Nematodes have demonstrated the ability to adapt and overcome resistance in *G. max*, and therefore new sources of SCN resistance are needed. *G. soja* has been shown to exhibit varying resistance to different SCN populations currently affecting crops in the United States (Kim et al., 2011; Zhang et al., 2016). A large portion of *G. soja* accessions is yet to be screened for resistance, and the investigation into the genetic mechanisms conferring resistance is still underway. So far, several studies have reported loci and candidate genes that might function differently from those thus far detected in *G. max* conferring SCN resistance using linkage mapping (Kim et al., 2011) and genome-wide association study (Zhang et al., 2016, 2017d), suggesting species-specific resistance mechanisms in *G. soja*. For example, two SCN-resistance QTLs (*cqSCN-006* on chr.15 and *cqSCN-007* on chr.18) identified in *G. soja* genotype PI468916 differ from the two major SCN resistance QTLs (*rhg1* on chr.18 or *Rhg4* on chr.8) identified in *G. max* (Kim et al., 2011), providing an alternative or additional sources of SCN resistance other than PI88788. On-going efforts in fine mapping of the underlying QTLs have identified SCN resistant candidate genes including SNAP gene *Glyma.15g191200* (Yu and Diers, 2017). Further molecular cloning experiments are expected to be made to increase our understanding of defense mechansims in *G. soja*.

On the other hand, extensive research on different races of SCN may increase our understanding of SCN resistance. Currently, the majority of studies has been mostly focused on resistance mechanisms to SCN HG type 0 (race3), which is prevalent in the central United States. However, few studies focused on other less-studied races, such as SCN HG type 2.5.7 (known as race 5), which is prevalent in the southeast United States. A recent study identified *G. soja* genotypes resistant to SCN HG type 2.5.7 (Zhang et al., 2016) and has identified novel QTLs in *G. soja* relating to HG type 2.5.7 resistance using genome-wide association study (Zhang et al., 2016). Further analysis of one of the resistant genotypes with RNA-seq data revealed a biologically-sound defense regulatory network involved in SCN resistance (Zhang and Song, 2017; Zhang et al., 2017b). Given the variability in virulence and rapid evolution of SCN populations, it is critical to develop soybean cultivars with broad-spectrum resistance to multiple SCN types. This can be achieved by identifying new sources of resistance and applying genome editing or gene stacking to develop soybean lines with broad resistance to SCN.

### Abiotic stress tolerance

Soil salinity is a growing challenge for today's crops. As the most important protein-providing crop, cultivated soybean is salt sensitive and has been cultured in well-plowed soil. The discovery of salt tolerance in *G. soja* was especially significant and has led to the first investigation of this trait in 1997, but only weak correlation has been found between the genetic markers chosen and the salt tolerance traits (Hu and Wang, 1997). Further investigation using randomly amplified polymorphism DNA markers revealed associations between 6 markers (OPF05-213, OPF19-4361, OPF19-1727, OPF19-14000-, OPF19-700, OPH02-1350) and salt tolerance in *G. soja*, but with no certainty of the genomic location of these markers (Zhang et al., 1999). The first discovered salt tolerance gene in *G. soja* was *Ncl2*, which has not been identified in *G. max* (Lee et al., 2009). Screening and sequencing of another wild soybean ecotype W05 have led to the discovery of a different salt-tolerant gene, *GmCHX1* (Qi et al., 2014), suggesting genotype-specific salt tolerance mechanisms. The mechanism of salt tolerance in a wild soybean genotype, Tongyu06311, was found to be regulated by amino acid and organic acid metabolism where compatible solutes were accumulated instead of relying on the consumption of ATP (Yang et al., 2017). Most recently, aquaporin gene *GmTIP2;1*, was found associated with salt tolerance in *G. soja* (Zhang et al., 2017a). Given the role of aquaporins in water transport, it is likely that more aquaporin alleles found in *G. soja* might be associated with salt stress tolerance.

The drought tolerance gene *GsWRKY20* from *G. soja* was recently identified and functionally validated by overexpressing it in soybean. The transgenic soybean exhibits increased yield, plant height, and root length over the non-transgenic plant in the same drought conditions. The mechanisms governing the drought tolerance is related to stomatal density and closure speed (Ning et al., 2017). This study provides promising solutions to farming in arid and semi-air environments.

A landscape genomics study of *G. soja* revealed candidate SNPs associated with varying environmental factors, including monthly precipitation, substrate sand percentage and substrate silt percentage (Anderson et al., 2016). These findings indirectly suggest these loci might play roles in certain abiotic stress tolerance, functional evaluation is needed for each of these genes before they can be considered for soybean crop improvement.

### Nutrition

Reduction of saturated fatty acid content of soybeans is highly desired due to its association with cardiovascular disease (Hu et al., 1997). A GWAS study on seed composition in *G. soja* revealed three new markers associated with palmitic acid levels, a saturated fatty acid, although increased resolution is needed to identify candidate genes. This result has led to the identification of a putative candidate genes, *Glyma.07G112100*, associated with biosynthesis of linoleic acid in *G. soja* (Leamy et al., 2017). Linoleic acid is a polyunsaturated fatty acid that is often partially hydrogenated and attributed to cardiovascular health. This recent discovery of a gene controlling both unwanted fatty acids has the potential to make marked improvements on the nutritional profile of the cultivated soybean. The same study of *G. soja* identified two candidate genes, *Glyma.14G121400* and *Glyma.16G068500*, which are associated with a saturated fatty acid, steric acid, and revealed a candidate gene associated with the “good” fatty acid, unsaturated oleic acid (Leamy et al., 2017). These candidate genes were reported with *G. max* gene IDs, although they were identified in *G. soja*. In addition, seed protein content of *G. soja* has been shown to be higher than *G. max* on average, likely due to selection on increased yield and oil content (Diers et al., 1992; Chen and Nelson, 2004; Leamy et al., 2017).

### Yield related traits

An investigation into the variation of early plant height in wild soybeans revealed significant differences among *G. soja* accessions (Chen and Nelson, 2006). This variation suggests that the trait has been under selection in the natural environment, and may have underlying genetic controls that can be introduced to cultivated soybean. However, characterizing *G. soja* height beyond 30 days is especially challenging due to the fragile vining nature of the species, limiting growth rate phenotyping to early stages. Nevertheless, early growth rates are important indicators of a plant's eventual success and yield (Chen and Wiatrak, 2011; Filho, 2015).

Yield has been strongly selected in cultivars, often making *G. max* superior in this trait. However, one study has identified a QTL in *G. soja* correlated with improved yield. QTL mapping of a cross population of *G. soja* with *G. max* lead to the discovery of a yield related QTL in *G. soja* on chromosome 14. This QTL is responsible for a 9.4% yield advantage and has been validated in two elite genetic backgrounds (Concibido et al., 2003).

## Perspectives and limitations

*G. soja* holds great potential to provide novel genes/alleles soybean and other legume species for crop improvement. A prerequisite for using *G. soja* to improve the agricultural potential of *G. max* is to dissect the genetic architecture underlying the traits of interest and uncover the molecular, physiological, and biochemical mechanisms involved. Some studies using GWAS and linkage mapping have facilitated the dissection of potentially useful traits, and an integration of these strategies coupled with investigation of gene expression (transcriptomes), protein expression (proteomics), and metabolite profiling (metabolomes) can be helpful to understand the mechanisms involved in phenotypic differences. High throughput sequencing technology and biotechnology (such as genome editing) can significantly facilitate novel gene discovery and transfer useful genes to soybean.

*G. soja* and *G. max* have the same chromosome number and can be easily crossed to create fertile hybrids, which make it possible to transfer useful genes from *G. soja* to *G. max* by traditional breeding practice. However, introgression of *G. soja* into *G. max* may result in linkage drag by bringing in unwanted parts of the genome together with the selected genes due to linkage disequilibrium. Linkage drag usually results in drags of unfavorable traits, such as reduced yield, apt to shatter, and lodging etc. This limitation can be resolved by the rapid progress of biotechnology, such as improved genetic transformation and cutting edge genome editing (e.g., Kim et al., 2017). The advanced breeding technologies, such as marker-assisted backcrossing (MABC) (e.g., Iftekharuddaula et al., 2011; Hasan et al., 2015) and genomic selection (e.g., Duhnen et al., 2017) can also improve the efficiency.

Thus far, *G. soja* has been used to determine the nature of domesticated traits in *G. max* (Li et al., 2014; Zhou et al., 2015b), while the associated genetic variation in *G. soja* has not been fully investigated. Knowledge of the genomic regions with selective sweeps in *G. soja* is helpful to understand the evolutionary mechanisms that produced and altered traits in *G. max* resulting from domestication, and can facilitate the use of *G. soja*-derived variations to improve soybean. Investigations of phenotypic variation should shift toward the screening for biotic and abiotic resistance under different environmental conditions and stressor regimes. Such a shift in research focus would produce results (and future directions) that are more relevant to current needs as they pertain to growing food needs and climate change. Although some studies have identified SCN-resistant *G. soja* accession by screening a small portion of *G. soja* collection at USDA germplasm (Kim et al., 2011; Zhang et al., 2016), a systematic screening is needed by collecting more ecotypes from many different natural populations, taking into account the observed association between the SCN resistance and *G. soja* origins. Meanwhile, it is critical to find out the genes/alleles associated with important traits in *G. soja* are species-specific by comparing them with those from cultivated soybean collections.

On the other hand, it is still time-consuming and labor intensive to measure agriculturally-important traits in *G. soja* because of its extensive lateral branching phenotype and indeterminate growth habit. The lack of high-throughput phenotyping technology poses challenges for research of genotype-phenotype interactions, especially those requiring large datasets (Houle et al., 2010). For example, phenotyping of root traits infected with root pathogens requires additional efforts and time. The measurement of SCN resistance in soybean roots requires at least 25 days of nematode inoculation (a life cycle of SCN), making it one of the most difficult traits to screen for.

Crop improvement by using wild soybean has been progressing, but is still lacking in some promising areas. Currently, very little work on the Soybean Mosaic Virus (SMV) has been reported in *G. soja*, a native host to the virus. *G. soja* plants infected by SMV have been observed in South Korea (Seo et al., 2009). More work is needed to develop SMV-resistant soybean varieties. In addition, the observed variation in early vigor traits in wild soybeans is also an understudied area with potential to improve the early success and eventual yield of soybean crop.

The chloroplast genome has been used in only a few studies with *Glycine*, and in those cases only to discuss diversity and domestication. Plastids regulate photosynthesis and the production of many metabolites; it would not be a great leap to assume that some of those attributes are (a) important to cultivation and (b) phenotypically found to vary in the wild population of soybean. The chloroplast genome of soybeans underwent multiple selection events, and diverged into two major haplotypes early on in domestication (Fang et al., 2016). Whole chloroplast genome assembly of *G. soja* compared to nine other *Glycine* species reported high conservation with no major rearrangements within *Glycine*. Phylogenetic topology is consistent between nuclear and plastid genomes, and an in-depth investigation into plastid genomic differences between *G. soja* and *G. max* may reveal attributes worth studying for crop improvement (Asaf et al., 2017).

Efforts should also be made to conserve the wild soybean populations, along with other crop wild relatives (Khoury et al., 2010; Castañeda-Álvarez et al., 2016). It has been shown that these diverse populations exhibit desired phenotypes and diverse genotypes that can be exploited for crop improvement. Conservation of these populations is required in order to continue benefiting from them. A recent study in Japan reported low risk of gene flow between genetically modified and wild soybeans (Goto et al., 2017). Soybeans self-fertilize, and therefore gene flow can easily be maintained by reducing seed transport and spillage and increasing the distance between crops and wild populations.

The natural populations of wild soybean are distributed in East Asia and Russia. Current research mainly uses a little over 1,000 genotypes from USDA collections. To fully investigate the natural variation of this species and identify useful resources, international collaborations between scientists from diverse disciplines are needed to collect and share germplasm, as well as share genomic resources. More findings from wild soybean are expected in the near future.

## Conclusion

The wild soybean has been the source of much advancement in crop improvement and aided in research related to the evolution of the soybean. The domestication history of soybean is now widely understood. Continued research and screening of more ecotypes for potentially useful traits in *G. soja* will help to reveal additional valuable genetic sources for genetic improvement of legumes. Collaborations and effort should be put toward finishing what has already begun—to link the discovery of candidate genes with studies on relevant gene regulatory networks and ultimately, the molecular and practical procedures for crop improvement. Lastly, the conservation of natural soybean populations needs to be promoted to ensure the continued existence of adaptable, wild traits that could later be used for crop improvement. International collaboration is needed to carry out goal-directed, comprehensive research of *G. soja* to make full use of this untapped genetic reservoir.

## Author contributions

JK, HZ, and B-HS wrote the manuscript and approved the final manuscript.

### Conflict of interest statement

The authors declare that the research was conducted in the absence of any commercial or financial relationships that could be construed as a potential conflict of interest.
